# ﻿The genus *Aridelus* Marshall (Hymenoptera, Braconidae, Euphorinae) from Japan, with description of a new species

**DOI:** 10.3897/zookeys.1092.73299

**Published:** 2022-04-06

**Authors:** Shunpei Fujie, Kaoru Maeto

**Affiliations:** 1 Osaka Museum of Natural History, Nagaikoen 1–23, Higashisumiyoshi, Osaka 546–0034, Japan Osaka Museum of Natural History Osaka Japan; 2 Laboratory of Insect Biodiversity and Ecosystem Science, Graduate School of Agricultural Science, Kobe University, Rokkodaicho 1–1, Nada, Kobe, Hyogo 657–8501, Japan Kobe University Kobe Japan

**Keywords:** *Aridelus*, host records, identification key, new species, stink bug parasitoids, taxonomy

## Abstract

Six Japanese species belonging to the genus *Aridelus* Marshall, 1898 (Hymenoptera, Braconidae) were recorded and photographed. Three species, *A.dubius* Belokobylskij, *A.egregius* Schmiedeknecht and *A.rufotestaceus* Tobias (= *Aridelusrufiventris* Luo & Chen **syn. nov.**), are new to Japan, and a new species, *A.rutilipoides***sp. nov.** is described. An identification key to the Japanese species of *Aridelus* is also provided. In addition, new host records are provided, i.e., *A.flavicans* Chao reared from *Homoeocerusunipunctatus* and *Riptortuspedestris* (Alydidae) and *A.rufotestaceus* reared from *Glauciassubpunctatus* (Pentatomidae). The Alydidae is a newly recorded host family of *Aridelus*.

## ﻿Introduction

The braconid subfamily Euphorinae is unique in attacking a wide range of host orders, including both larvae and adult insects (Stigenberg et al. 2015). Its adult morphology varies greatly, probably due to adaptive evolution, which enables it to utilize a variety of free-living host insects (Shaw 1985, 1988; Maeto 2018).

The genus *Aridelus* Marshall, 1887 has an aberrant morphology, that is, the entirely areolate mesosoma and the elongated tubular first metasomal tergite. Using a petiolated metasoma with a short ovipositor, females lay eggs into nymphs or adults of heteropteran stink bugs (Shaw 1985; Maeto and Kudo 1992; Shaw et al. 2001). They are hitherto known to be solitary koinobiont endoparasitoids of the families Acanthosomatidae, Pentatomidae, Plataspidae, and Scutelleridae (Shaw et al. 2001). Although more than 40 species of *Aridelus* are known worldwide (Yu et al. 2016), only two species, *A.elasmuchae* Maeto & Kudo, 1992 and *A.flavicans* Chao, 1974, have been recorded in Japan (Maeto and Kudo 1992; Konishi and Maeto 2000).

In our study of Japanese Euphorinae, we identified six species of *Aridelus*, that is, *A.dubius* Belokobylskij, 1981, *A.egregius* (Schmiedeknecht, 1907), *A.elasmuchae*, *A.flavicans*, *A.rufotestaceus* Tobias, 1986, and *A.rutilipoides* sp. nov. In this study, all Japanese species are photographed, a new species is described, and an identification key to the Japanese species is provided. In addition, new host records of *A.flavicans* and *A.rufotestaceus* are presented herein.

## ﻿Materials and methods

The specimens examined were deposited in Kanagawa Prefectural Museum of Natural History, Odawara, Japan (**KPMNH**), Laboratory of Entomology, Faculty of Agriculture, Meijo University, Nagoya, Japan (**MUNJ**), Insect Museum, National Agriculture and Food Research Organization, Tsukuba, Japan (**NARO**), National Science Museum, Tokyo, Japan (**NSMT**), Osaka Museum of Natural History, Osaka, Japan (**OMNH**), Taiwan Agricultural Research Institute, Taichung, Taiwan (**TARI**), and Zoological Institute, Russian Academy of Sciences, St. Petersburg, Russia (**ZISP**). MsT. and LT. refer to a Malaise trap and a light trap, respectively. Besides six Japanese species, two females of *A.rutilipes* Papp, 1965 from Taiwan [2♀, Lixing Industry Road, Renai Township, Nantou Country, 8.X.2015, So Shimizu leg. (TARI)] were also examined.

Morphological observation was conducted using a stereoscopic microscope (SMZ800N, Nikon, Tokyo, Japan). The specimens were photographed using a digital microscope (VHX-1000, Keyence, Osaka, Japan) with a 10–130× lens. Multi-focus photographs were stacked in the software associated with the Keyence System. The figures were edited using Microsoft PowerPoint 2019.

The morphological terminology used is mostly based on van Achterberg (1988, 1993). OOL, OD, and POL refer to the ocellar-ocular line, the diameter of the posterior ocellus, and the postocellar line, respectively.

## ﻿Taxonomic accounts

### ﻿Genus Aridelus

Taxon classificationAnimaliaHymenopteraBraconidae

Marshall, 1887

A2CB99A0-0E6C-57CE-9142-0E15ED520786

Aridelus Marshall, 1887: 66; Papp 1965: 181; Shenefelt 1969: 11; Shaw 1985: 309; Chou 1987: 21; Chen and van Achterberg 1997: 11; Belokobylskij 2000: 362.Synonyms are presented in Shenefelt (1969) and Shaw (1985).

#### Type species

**(by monotypy).***Aridelusbucephalus* Marshall, 1887.

#### Diagnosis.

***Head*** transverse; antenna filiform or moniliform, with 18 segments, its terminal segment with an apical spine; maxillary palp with 6 segments; labial palp with 4 segments; occipital carina complete or absent mediodorsally for a long distance, rarely completely absent, ventrally joining hypostomal carina; frons punctate or smooth with a median carina extending to frontal ocellus; face wider than clypeus in female; lower clypeal margin usually indented medially, rarely rounded; malar suture usually absent; mandibles overlapping each other; mesonotum, mesopleuron, and propodeum mostly areolate; petiolar notch extending nearly to mesocoxal insertions; parastigma large; vein 1-SR of fore wing absent to rarely shortly present and thickened; vein 3-SR of fore wing absent to distinctly present; vein 1-R1 of fore wing short; end of vein SR1 of fore wing much closer to pterostigma than to apex of wing; vein r-m of fore wing present; veins SR and 2-M of hind wing present, darkly pigmented; first metasomal tergite about 3/4 times as long as remainder of metasoma and completely fused ventrally; third tergite nearly reaching end of metasoma, following segments hidden; second and third tergites ventrally overlapping, without lateral fold; ovipositor and its sheath shortly exposed.

#### Distribution.

Cosmopolitan and the most diverse in tropical regions (Yu et al. 2016).

#### Bionomics.

Endoparasitoids of nymphs and adults of Acanthosomatidae, Pentatomidae, Plataspidae, and Scutelleridae (Shaw et al. 2001), and of Alydidae (present study). Usually diurnal, but a few species were collected at night in light traps (e.g., *A.dubius* in the present study).

### ﻿Key to the Japanese species of the genus *Aridelus* Marshall

**Table:** 

1	Median carina of frons only weakly developed (Fig. 2B); occipital carina absent dorsally; inner margins of eyes not parallel, distinctly convergent ventrally (Fig. 2B); hind femur distinctly stout, 3.0× longer than wide (Fig. 2E); mesosoma black, head and metasoma except for first metasomal tergite black to dark brown (Fig. 2A); body length shorter, 2.7–3.7 mm [vein m-cu of fore wing postfurcal to interstitial (Fig. 2D)]	***A.egregius* (Schmiedeknecht)**
–	Median carina of frons distinct (Figs 1B, 3B, 4B, 7B); occipital carina complete; inner margins of eyes almost parallel or weakly convergent (Figs 1B, 3B, 4B, 7B); hind femur comparatively slender, at least 4.0× longer than wide (Figs 1G, 7H); body colour variable, but not entirely black (Figs 1A, 3A, 4A, 5A, 7A); body length longer, 3.6–6.5 mm	**2**
2	Head yellow, yellowish red, yellowish brown or reddish brown (Figs 3C, 4C, 5B, C); fore wing without distinct fuscous bands (Figs 3A, 4A, 5A) .	**3**
–	Head black (Figs 1C, 7C); fore wing with two distinct fuscous bands (Figs 1F, 7G)	**5**
3	First metasomal tergite pale yellow, distinctly contrast to blackish or dark brownish second and following tergites (Fig. 3A); fore wing fuscous in distal half (Fig. 3A); mesosoma entirely black (Fig. 3A) [head yellowish brown; vein m-cu of fore wing postfurcal, rarely interstitial; body length 3.6–5.0 mm]	***A* . *elasmuchae* Maeto & Kudo**
–	First metasomal tergite yellowish red to reddish brown, not distinctly contrasting to second and following tergites (Figs 4F, 5A); fore wing hyaline or slightly infuscated medially (Fig. 4A, 5A); colour of mesosoma variable	**4**
4	Vertex punctate and without transverse rugae (Fig. 4C); scutellum with median smooth area (Fig. 4E); mesosoma yellow to yellowish brown, not distinctly contrast to head and metasoma (Fig. 4A); penultimate segment of ♀ antenna 1.1–1.6× longer than wide (Fig. 4D) [vein m-cu of fore wing postfurcal to antefurcal; body length 4.1–5.3 mm]	***A.flavicans* Chao**
–	Vertex punctate, often transversely rugose (Fig. 5B, C); scutellum uniformly areolate, without median smooth area (cf. Fig. 7F); mesosoma reddish brown to black, usually distinctly contrasting to reddish head and metasoma (Figs 5D, E); penultimate segment of ♀ antenna 1.8–2.0× longer than wide [body length 4.5–5.7 mm]	***A.rufotestaceus* Tobias**
5	Penultimate segment of antenna 1.2–1.3 times as long as its width (Fig. 7E); antenna yellowish brown basally (Fig. 7D), darkened towards apex (Fig. 7E); face finely punctate laterally, transversely punctate-rugose medially; malar suture indistinct or absent; vein m-cu of fore wing distinctly antefurcal (Fig. 7G); apical hyaline area of fore wing comparatively large, almost reaching apex of marginal cell (Fig. 7G); hind femur slenderer, 4.6–5.2× longer than wide (Fig. 7H) [body length 5.8–6.5 mm]	***A.rutilipoides* sp. nov.**
–	Penultimate segment of antenna 1.6–1.7× longer than wide (Fig. 1E); antennal segments entirely reddish brown (Fig. 1D, E); face fairly finely punctate; malar suture distinct; vein m-cu of fore wing slightly postfurcal to interstitial (Fig. 1F); apical hyaline area of fore wing comparatively small, not reaching apex of marginal cell (Fig. 1F); hind femur stouter, 4.1–4.3× longer than wide (Fig. 1G) [body length 5.1–6.1 mm]	***A.dubius* Belokobylskij**

### ﻿ Aridelus dubius

Taxon classificationAnimaliaHymenopteraBraconidae

Belokobylskij, 1981

5A9D6921-27F9-531D-A098-1A1C276F6C83

Fig. 1A–G

Aridelus dubius Belokobylskij, 1981: 44. [Type locality: Russia]

#### Material.

**Japan Honshû**: 1♀, Niigata Pref., Myoukou City, Suginosawa, Sasagamine, 36-52N/138-4E, about 1200–1335 m alt., 18.IX.2013, S. Shimizu leg. (OMNH); 1♀, same data except 36-52-2N/138-4-42E, about 1300 m alt., 14.IX.2013, LT. (NARO); .1♀, Hiroshima Pref., Shôbara City, Saijô Town, Mts. Hiba, Tachieboshi Parking Lot, 30–31.VII.2019, LT., S. Shimizu leg (OMNH).

#### Description.

**Figure 1.**
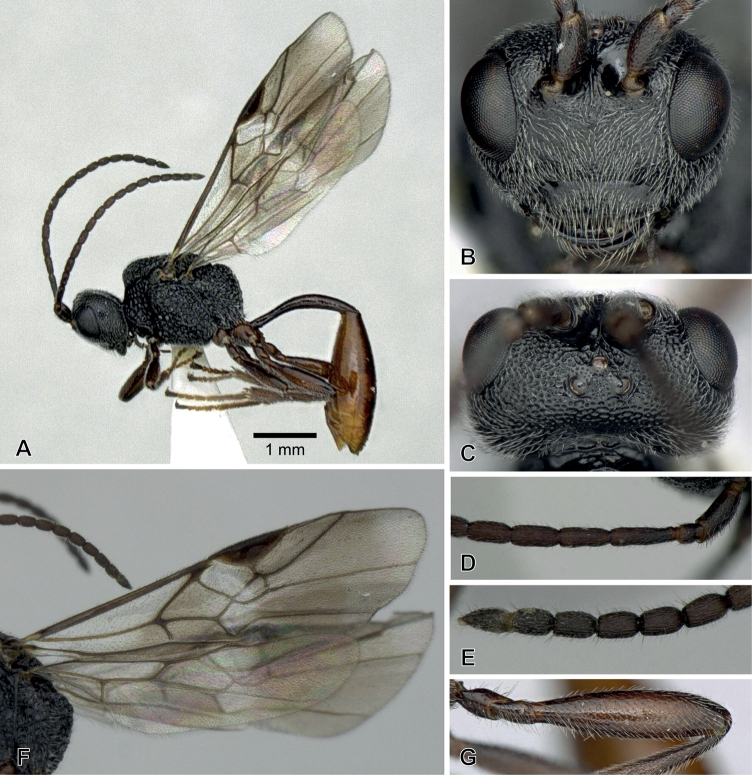
*Aridelusdubius* Belokobylskij, 1981, ♀ from Niigata Prefecture, Japan **A** habitus **B** head, frontal view **C** head, dorsal view **D** basal antennal segments **E** apical antennal segments **F** wings **G** hind femur.

**Females** (*N* = 3) (Fig. 1). Body length 5.1–6.1 mm.

***Head*** (Fig. 1B, C, D, E). Width of head 2.0–2.2× median length and 1.0× width of mesonotum. Length of eye 1.1–1.3× length of temple in dorsal view. OOL / OD = 4.0–4.1. POL / OD = 1.7. Vertex and temple densely punctate. Occipital carina complete. Frons smooth and shiny, with a distinct median carina. Face 1.8× as wide as high; finely punctate. Intertentorial distance / tentorio-ocular distance = 0.4–0.5×. Clypeus finely punctate, slightly concave medially, without apical teeth. Length of malar space 0.4–0.5× eye height. Malar suture distinct. Antenna filiform with 18 segments; 3^rd^ segment 3.8–4.0× longer than wide and 1.3× longer than 4^th^ one; penultimate one 1.6–1.7× longer than wide.

***Mesosoma*.** Mesosoma areolate, 1.3× as long as high. Scutellum without median smooth area.

***Wings*** (Fig. 1F). Fore wing 4.4–4.7 mm in length, 1-R1 / length of pterostigma = 1.3–1.4, r / 3-SR = 2.3–3.5, m-cu slightly postfurcal. Hind wing with 1r-m / 2-SC+R = 0.7–0.9.

***Legs*** (Fig. 1G). Hind leg: femur 4.1–4.3× longer than wide, length of femur: tibia: basitarsus = 1: 1.3: 0.6.

***Metasoma*.** Metasoma smooth and polished. First metasomal tergite 6.0× longer than its apical width. Hypopygium truncated and excised apically. Ovipositor sheath hardly exserted beyond apex of metasoma.

***Colour*.** Black. Palpi, antenna entirely, mandible, tegula, legs except for telotarsus and first metasomal tergite dark reddish brown; remainder of metasoma reddish brown, telotarsus and veins dark brown; pterostigma pale in basal 1/5 or faintly pale basally. Fore wing hyaline with two fuscous bands. Hind wing with a fuscous band in its apical third.

#### Distribution.

Japan (Honshû: Niigata and Hiroshima Prefectures); Russian Far East (Belokobylskij 1981, 2000).

#### Hosts.

Unknown.

#### Remarks.

This species was described with only the male holotype available. The Japanese specimens mostly agree well with the original description (Belokobylskij 1981) and run in the key by Belokobylskij (2000) to *A.dubius*. The redescription of this species based on Japanese female specimens is represented here.

This species resembles *A.rutilipes* Papp described from Taiwan (Fig. 6) but differs in having the distinct malar suture (absent in *rutilipes*), the palpi dark reddish brown (light reddish brown in *rutilipes*), the apical hyaline area of the fore wing comparatively small, not reaching the apex of marginal cell (Fig. 1F) and the apico-posterior edge of the fore wing (in *rutilipes* comparatively large, almost reaching the apex of marginal cell and reaching the apico-posterior edge (Fig. 6A)), and the metasoma reddish brown to dark reddish brown (Fig. 1A) (dark brown in *rutilipes* (Fig. 6A)).

### ﻿ Aridelus egregius

Taxon classificationAnimaliaHymenopteraBraconidae

(Schmiedeknecht, 1907)

3FCFB3F7-6DA3-5436-A9F5-524AB9E09756

Fig. 2A–E

Helorimorpha egregia Schmiedeknecht, 1907: 523. [Type locality: Germany]
Aridelus egregia (Schmiedeknecht): Muesebeck 1936: 6; Shenefelt 1969: 12.
Aridelus nigricans Chao, 1974: 455. Syn. by Belokobylskij 2000: 366. [Type locality: China]
Aridelus destitutus Chou, 1987: 26. Syn. by Chen and van Achterberg 1997: 17. [Type locality: Taiwan]

#### Material.

**Japan Honshû**: 1♀, Tôkyô Pref., Chiyoda Ward, Imperial Palace, Fukiagegyoen, Kajuen, 14–21.X.2009, MsT. (NSMT); 1♀, Fukui Pref., Tsuruga City, Marsh of Nakaikemi, 19.IX–16.X. 2016, MsT., A. Noishiki leg. (OMNH). **Kyûshû**: 2♀♀, Ôita Pref., Mt. Sobo, 1600–1750 m alt., 27.IX.1979, K. Maetô leg. (NARO). **Korea** 1♂, Kyongsangpuk-do, Mt. Sudo, 1000 m alt., 13–14.VII.1971, K. Yamagishi leg. (MUNJ). **Taiwan** 2♂♂, Nantou Country, Renai Township, 2.V.2015, S. Fujie leg. (TARI).

**Figure 2.**
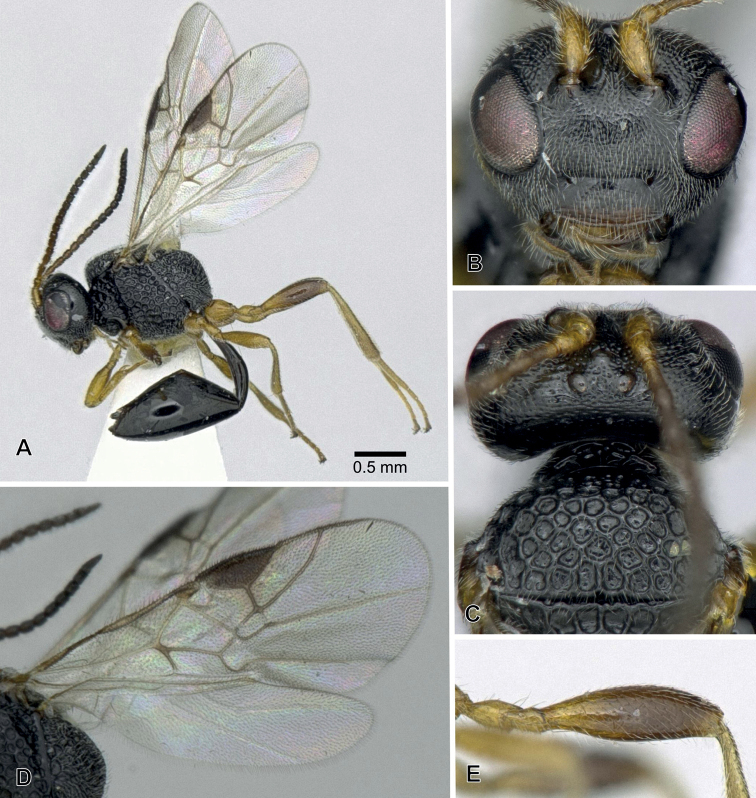
*Aridelusegregius* Schmiedeknecht, 1907, ♀ from Fukui Prefecture, Japan **A** habitus **B** head, frontal view **C** head and mesoscutum, dorsal view **D** wings **E** hind femur.

#### Distribution.

Japan (Honshû: Tôkyô and Fukui Prefectures; Kyûshû: Ôita Prefecture); Western Palaearctic region; China, Korea, Russian Far East, Taiwan (Yu et al. 2016; Lee at al. 2017). New to Japan.

#### Hosts.

No host records are available in Japan, while Pentatomidae (*Aelia*, *Dolycoris*, *Eurydema*, *Holcostethus*, *Palomena*) (Dupuis 1952; Tobias 1986), Plataspidae (*Coptosoma*) (Capek and Davidova-Vilimova 1978), and Scutelleridae (*Eurygaster*) (Tobias 1986) are known host insects.

### ﻿ Aridelus elasmuchae

Taxon classificationAnimaliaHymenopteraBraconidae

Maeto & Kudo, 1992

F20BFEE8-3903-5540-8883-AAE82C774059

Fig. 2A–C

Aridelus elasmuchae Maeto & Kudo, 1992: 78. [Type locality: Japan]

#### Material.

**Japan Hokkaidô**: 1♀, ***holotype***, Nopporo, 15.VII.1986, S. Kudô leg. (NARO); 2♂♂, Sapporo City, Hitsujigaoka, 20–27.VI.2011, MsT., K. Konishi leg. (OMNH). 2♀♀ and 2♂♂, same data except 27.VI–4.VII.2011 (OMNH); 1♀, Kumaishi Town, Ken’ichi-gawa, Iwafuchi-zawa, 15–20.VI.1995, MsT., Y. Itô & T. Itô leg. (NARO). **Honshû**: 1♀, Aomori Pref., Mt. Iwaki, 28.IX.1983, M. Miyazaki leg. (NARO); 9♀♀ 7♂♂, Miyagi Pref., Minamisanriku Town, Hinokuchi, 5.VI–12.VII.2015, MsT., H. Yamazaki & S. Fujie leg. (OMNH); 2♂♂, same data except 28.IX–6.XI.2015 (OMNH); 1♀, Miyagi Pref., Minamisanriku Town, Mt. Tatsugane, 5.VIII.2016, S. Fujie leg. (OMNH); 1♂, Toyama Pref., Toyama City, Arimine, Jurodani, 1120 m alt., 7–14.VII.2009, MsT., M. Watanabe leg. (KPMNH); 1♀, Hyôgo Pref., Kami Town, Niiya, 20.VI–11.VII.2015, MsT., S. Fujie leg. (OMNH); 1♀, Hyôgo Pref., Kami Town, Niiya, 15.VI–14.VII.2013, MsT., S. Fujie & M. Itô leg. (OMNH). **Kyûshû**: 1♂, Kumamoto Pref., Izumi Vil., Mt. Hakuchô, 10.VII.1978, K. Ohara leg. (NARO).

**Figure 3.**
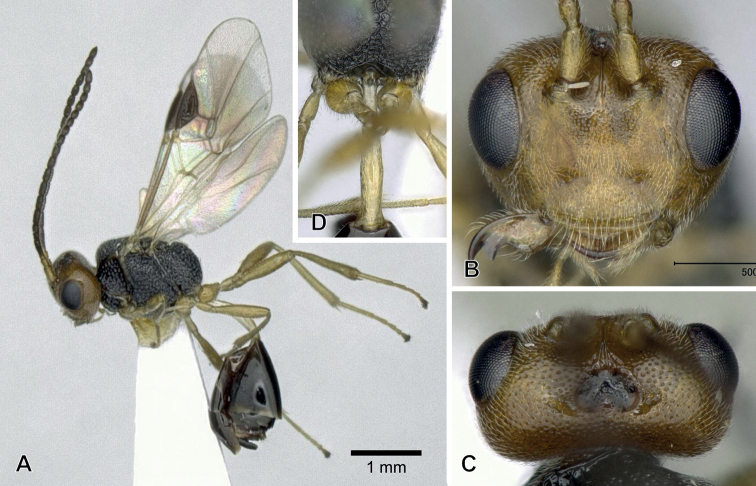
*Arideluselasmuchae* Maeto & Kudo, 1992, ♀ from Hyôgo Prefecture, Japan **A** habitus **B** head, frontal view **C** head, dorsal view **D** first metasomal tergite, dorsal view.

#### Distribution.

Japan (Hokkaidô; Honshû: Aomori, Miyagi, Tochigi, Toyama, Hyôgo and Tottori Prefectures; Shikoku: Ehime Prefecture; Kyûshû: Ôita Prefecture) (Maeto and Kudo 1992; Takahashi and Shiraishi 2018; Fujie and Katayama 2020; present study); Russian Far East (Yu et al. 2016).

#### Hosts.

*Elasmuchaputoni* Scott, 1874 (Acanthosomatidae) (Maeto and Kudo 1992).

### ﻿ Aridelus flavicans

Taxon classificationAnimaliaHymenopteraBraconidae

Chao, 1974

DCFE1602-BB50-57AD-BAD1-72D9C945BF0A

Fig. 4A–F

Aridelus flavicans Chao, 1974: 455; Chou 1987: 23; Luo and Chen 1994: 484; Chen and van Achterberg 1997: 16. [Type locality: China]
Aridelus guizhouensis Luo, 1985: 203. Syn. by Luo and Chen 1994. [Type locality: China]

#### Material.

**Japan Honshû**: 1♀, Aomori Pref., Aomori City, Yokouchi-Yaegiku, 11.IX.1993, T. Ichita leg. (NARO); 1♂, Tôkyô Pref., Hachiôji City, Minamiôsawa, Tôkyô Metropolitan University, 10.VIII.2010, N. Kikuchi leg. (OMNH); 1♀, Kyôto Pref., Yawata City, Yawatahayashinomoto, collected as a host larva of *Homoeocerusunipunctatus* feeding on *Puerarialobata* on 12.VII.2021, cocoon formed on 23.VII.2021, and emerged on 29.VII.2021, S. Fujie leg. (OMNH); 2♀♀, Nara Pref., Yamatokôriyama City, Yamadachô, Nara-gakuen, 8.VII.2017, R. Itô leg. (OMNH); 1♀, Nara Pref., Yamatokôriyama City, Yamadachô, 6.IX.2016, R. Itô leg. (OMNH); 3♀♀, Nara Pref., Yamatokôriyama City, Yamadachô, Yata-kyûryô, about 135 m alt., 8.IX.2018, R. Itô leg. (OMNH); 1♀, same data except 13.IX.2018 (OMNH); 1♀, Nara Pref., Uda City, Haibarahagihara, Torimiyama-kôen, about 585 m alt., 20.VIII.2018, R. Itô leg. (OMNH); 1♀, Ôsaka Pref., Takatsuki City, Settsukyô, 24.IX.2017, S. Fujie leg. (OMNH); 6♀♀ and 1♂, Ôsaka Pref., Habikino City, Shakudo, 3.VIII.2020, S. Fujie leg. (OMNH); 2♂♂, same data except 22.VIII.2020 (OMNH); 2♀♀, same data except 2.VIII.2021 (OMNH); 1♀, Hyôgo Pref., Kawanishi City, collected as a host adult of *Riptortusclavatus* feeding on *Phaseolusvulgaris* on 24.VIII.2011, cocoon formed on 13.IX.2011, and emerged on 27.IX.2011, I. Hikino leg. (NARO); 1♂, same locality, host, collector and date of cocoon formation, collected on 29.VIII.2011 and emerged on 27.IX.2011 (NARO); 1♂, same locality, host, collector and date of cocoon formation, collected on 31.VIII.2011 and emerged on 28.IX.2011 (NARO); 1♀, Hyôgo Pref., Kôbe City, Nada Ward, Nadamaruyama Park, 23.VI.2019, M. & S. Fujie leg. (OMNH); 1♀, Hyôgo Pref., Asago City, Tataragi, 185 m alt., 30.VII. 2016, T. Tokuhira leg. (KPMNH); 1♀, Tottori Pref., Inaba Prov., Ketaka, Tsuyutani, alt. 20 m, 8.VIII.1964, H. Aoki leg. (OMNH); 1♀, Hiroshima Pref., Tôjô Town, Taishaku, 9.VIII.1978, K. Maetô leg. (NARO). **Kyûshû**: 1♀, Fukuoka Pref., Mt. Tachibana, 22.IX.1979, K. Maetô leg. (NARO); 1♀, Ôita Pref., Mt. Sobo, 800–900 m alt., 22.VII.1978, K. Maetô leg. (NARO); 1♀, Ôita Pref., Yufuin Town, Shimoyunohira, 1.IX.1991, M. Hiratate leg. (NARO).

**Figure 4.**
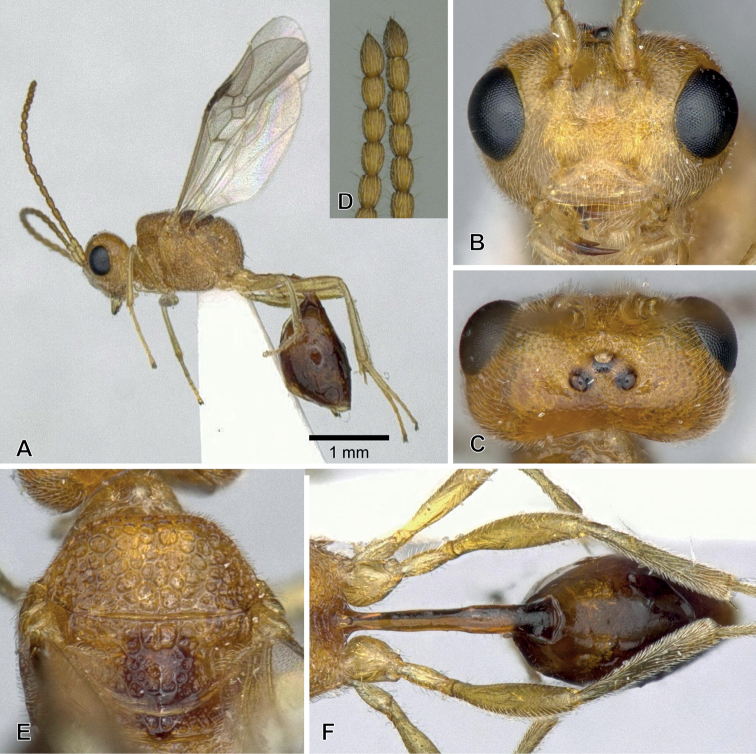
*Aridelusflavicans* Chao, 1974, ♀ from Tôkyô Prefecture (except for **D** from Ôsaka Prefecture), Japan **A** habitus **B** head, frontal view **C** head, dorsal view **D** apical part of antennae **E** mesoscutum and scutellum, dorsal view **F** metasoma, dorsal view.

#### Distribution.

Japan (Honshû: Aomori, Tôkyô, Nara, Ôsaka, Hyôgo, Tottori and Hiroshima Prefectures; Kyûshû: Fukuoka and Ôita Prefecture) (Konishi and Maeto 2000; present study); China, Russian Far East, Taiwan (Yu et al. 2016).

#### Hosts.

*Homoeocerusunipunctatus* (Thunberg, 1783) (Alydidae) feeding on *Puerarialobata* (Fabaceae) and *Riptortuspedestris* (Linnaeus, 1758) (Alydidae) on *Phaseolusvulgaris* (Fabaceae) (both new records). The family Alydidae is the first record of the host of the genus *Aridelus*.

#### Remarks.

The female specimens examined agree well with the redescription by Chou (1987) and that in the key by Chen and van Achterberg (1997); they differ slightly by having the body length 4.1–5.3 mm (4.2–5.1 mm in the previous redescriptions) and the penultimate antennal segment 1.1–1.6× longer than wide (1.2–1.6× in the redescriptions).

### ﻿ Aridelus rufotestaceus

Taxon classificationAnimaliaHymenopteraBraconidae

Tobias, 1986

B88ADBDE-BC96-5CDA-A41E-EBE823702FB8

Fig. 5A–I

Aridelus rufotestaceus Tobias, 1986: 229 (English translation: 399); Chen and van Achterberg 1997: 18; Shaw et al. 2001: 132. [Type locality: Russia]
Aridelus rufiventris Luo & Chen, 1994: 483; Chen and van Achterberg 1997:18. Syn. nov. [Type locality: China]

#### Material.

**Russia** 1♀, ***holotype***, Lazarevskoe, Sochi, forest along rivulet, 14.IX.1981, V. Tobias leg. (ZISP). **Japan Honshû**: 1♂, Shizuoka Pref., Shizuoka City, Shimizu Ward, Muramatsu, collected as a host adult of *Glauciassubpunctatus* on 14.XII.2018, recognized a cocoon on 23.I.2019, and emerged on 8.II.2019, K. Itoyama & A. Tsunashima leg. (OMNH); 1♂, Toyama Pref., Toyama City, Arimine, Inonedani, 1120 m alt., 15–22.IX.2009, MsT., M. Watanabe et al. leg. (KPMNH); 1♀, Mie Pref., Taiki Town, Nishiki, 9–21.X.2007, MsT., M. Nakaseko leg. (MUNJ); 1♂, Kyôto Pref., Yawata City, Morigaito, left bank of Kidu River, 17.VI.2018, S. Fujie leg. (OMNH); 1♀, Nara Pref., Uda City, Haibarahagihara, Torimiyama-kôen, about 585 m alt., 20.VIII.2018, R. Itô leg. (OMNH). **Hachijôjima Is.**: 2♂♂, Eigo, 2.VI.1964, Y, Hirashima & M. Shiga leg. (NARO). **Kyûshû**: 1♂, Kumamoto Pref., Izumi Vil., Gokanosô, 1.VIII.1981, H. Kurokawa leg. (OMNH). **Yakushima Is.**: 1♀, Kankake, 25.VIII–28.IX.2007, MsT., T. Yamauchi leg. (KPMNH); Mt. Aiko, 8–28.VI.2007, MsT., T. Yamauchi leg. (KPMNH); 1♀, same data except 29.VII–25.VIII.2007. (KPMNH); 1♀, same data except 28.IX–2.XI.2007. (KPMNH). **Korea** 1♂, Kyongsangpuk-do, Mt. Sudo, 700 m alt., 9–12.VII.1971, K. Yamagishi leg. (MUNJ).

**Figure 5.**
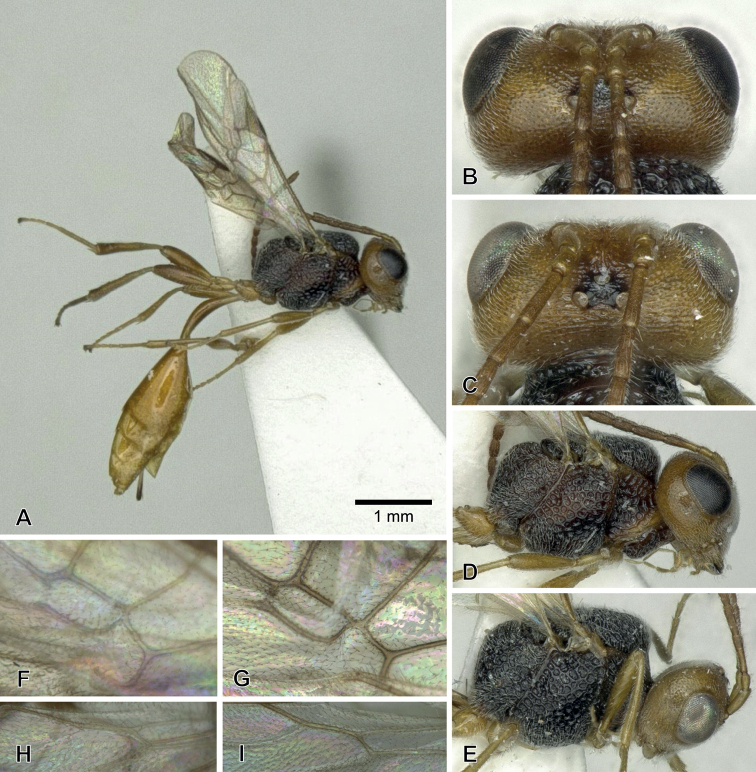
*Aridelusrufotestaceus* Tobias, 1986, ♀ from Yakushima Is. (A, B, D, F, H) and ♂ from Toyama Prefecture (C, E, G, I) **A** habitus **B, C** head, dorsal view **D, E** head and mesosoma, lateral view **F, G** second submarginal cell and vein m-cu of fore wing **H, I** vein 1r-m and 2-SC+R of hind wing.

#### Distribution.

Japan (Honshû: Shizuoka, Toyama, Mie and Kyôto Prefecture; Hachijôjima Is.; Kyûshû: Kumamoto Prefecture; Yakushima Is.); China, Georgia, Korea, Italy, Russian Far East (Yu et al. 2016; Lee et al. 2017). New to Japan.

#### Hosts.

*Glauciassubpunctatus* (Walker, 1867) (a new record) and *Nezaraviridula* (Linnaeus, 1758) (Shaw et al. 2001) (both Pentatomidae).

#### Remarks.

Chen and van Achterberg (1997) indicated the differences between *A.rufiventris* Luo & Chen and *A.rufotestaceus* Tobias from only one specimen of each taxon in the sculpture of the vertex, the condition of vein m-cu of the fore wing, the relative length of vein 1r-m of the hind wing, and the colour of the mesosoma, as shown in Suppl. material 1: Table S1. However, these characters could not separate the Japanese specimens into the two species (Suppl. material 1: Table S1). These are most likely intraspecific variations. Hence, *A.rufiventris* is considered a junior synonym of *A.rufotestaceus*.

### ﻿ Aridelus rutilipoides sp. nov.

Taxon classificationAnimaliaHymenopteraBraconidae

1B1E90F3-A09A-5DD4-9DCF-D6746623D978

http://zoobank.org/7A58CF41-B558-41CA-A5E6-DBDF8D66D1F2

Fig. 7A–I

#### Type material.

***Holotype***, ♀, “(JAPAN) Nagano Pref., Ueda City, Sugadaira-kougen, Tsukuba Univ., 36-31N/138-20E, about 1300 m alt., 13 IX 2013 (sweeping), Sou Shimizu leg.” (OMNH). ***Paratypes***: 1♀, Hokkaidô Pref., Sapporo City, Teine Ward, Mt. Teine, 18.IX.2013, S. Fujie leg. (OMNH); 1♀, Tochigi Pref., Nikkô, 13.X.1986, M. Miyazaki leg. (NARO); 1♀, Nagano Pref., Shimashima-dani, 1300–1600 m alt., 26.VIII.1978, K. Maetô leg. (NARO); 1♀, Tokushima Pref., Ichiu Vil., Mt. Tsurugi, 15.X.1980, Y. Shôno leg. (NARO).

**Figure 6.**
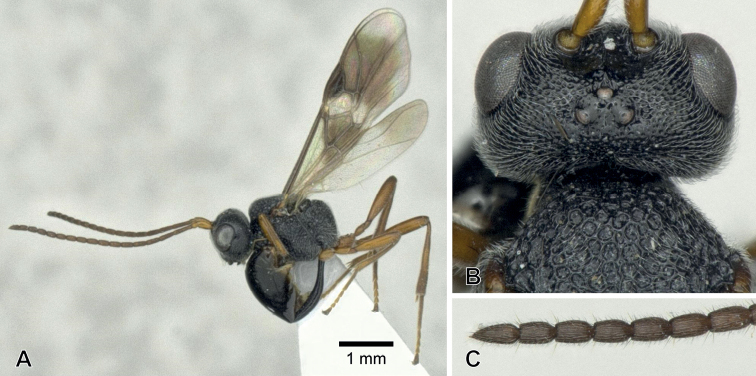
*Aridelusrutilipes* Papp, 1965, ♀ from Taiwan **A** habitus **B** head, dorsal view **C** apical antennal segments.

#### Etymology.

Named after its similarity to *A.rutilipes* Papp. The Latin suffix –“oides” taken from Greek means similar but not the same.

#### Description.

**Figure 7.**
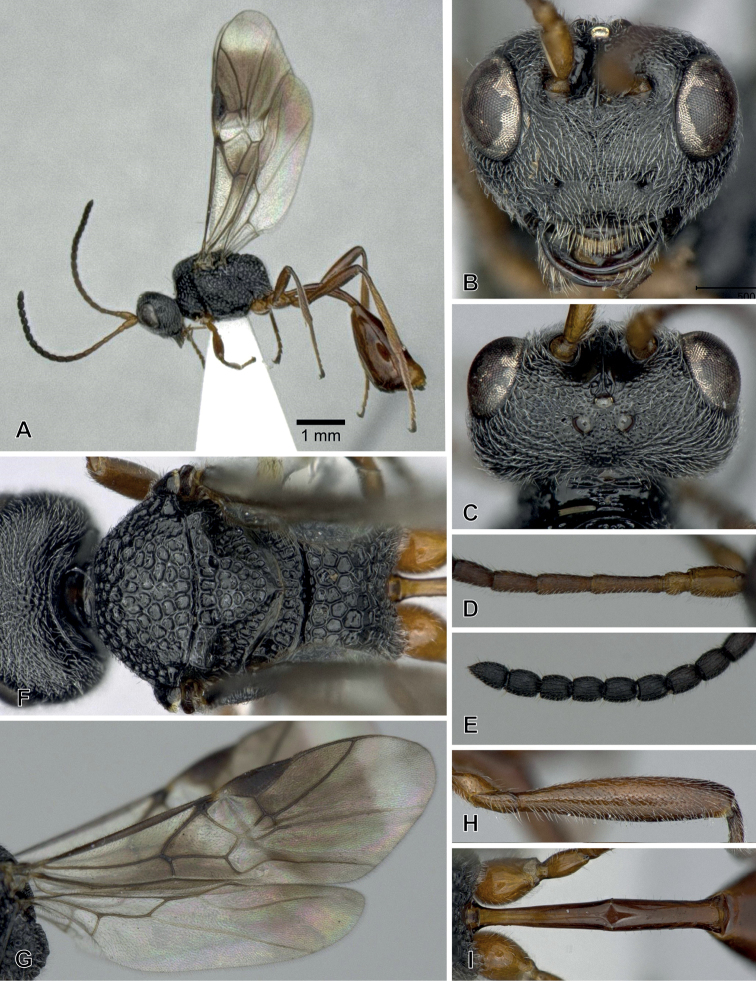
*Aridelusrutilipoides* sp. nov., ♀ holotype **A** habitus **B** head, frontal view **C** head, dorsal view **D** basal antennal segments **E** apical antennal segments **F** mesosoma, dorsal view **G** wings **H** hind femur **I** first metasomal tergite, dorsal view.

**Female *holotype*** (Fig. 7). Body length 6.5 mm.

***Head*** (Fig. 7B, C, D, E). Width of head 2.0× median length and 1.1× width of mesonotum. Length of eye 1.2× length of temple in dorsal view. OOL / OD = 4.1. POL / OD = 1.7. Vertex and temple densely punctate. Occipital carina complete. Frons smooth and shining, with a distinct median carina. Face 1.9× as wide as high; finely punctate laterally, transversely punctate-rugose medially. Intertentorial distance / tentorio-ocular distance = 1.7. Clypeus finely punctate, slightly concave medially, without apical teeth. Length of malar space 0.60× eye height. Malar suture indistinct. Antenna filiform with 18 segments; 3^rd^ segment 3.5× longer than wide and 1.2× longer than 4^th^ one; penultimate one 1.3× longer than wide.

***Mesosoma*** (Fig. 7F). Mesosoma areolate, 1.3× as long as high. Scutellum without median smooth area.

***Wings*** (Fig. 7G). Fore wing 5.0 mm in length, 1-R1 / length of pterostigma = 1.3, r / 3-SR = 2.4, m-cu distinctly antefurcal. Hind wing with 1r-m / 2-SC+R = 0.8.

***Legs*** (Fig. 7H). Hind leg: femur 5.2× longer than wide, length of femur: tibia: basitarsus = 1: 1.3: 0.6.

***Metasoma*** (Fig. 7I). Metasoma smooth and polished. First metasomal tergite 5.9× longer than its apical width. Hypopygium truncated and excised apically. Ovipositor sheath hardly exserted beyond apex of metasoma.

***Colour*.** Black. Antenna basally, mandible medially, palpi, tegula, legs except for telotarsus, metasoma reddish brown; mandible basally and apically, veins, telotarsus dark brown, antenna gradually darkened towards apex; pterostigma pale in basal quarter. Fore wing hyaline with two fuscous band. Hind wing with a fuscous band in apical third.

**Variation in females.** Body length 5.8–6.5 mm. Length of eye 1.2–1.4× length of temple in dorsal view; OOL / OD = 3.5–4.1. POL / OD = 1.5–1.9. Face 1.7–1.9× as wide as high. Intertentorial distance / tentorio-ocular distance = 1.7–1.9. Length of malar space 0.5–0.6× eye height. Malar suture indistinct or absent. Third antennal segment 3.5–4.0× longer than wide and 1.2–1.4× longer than 4^th^ one; penultimate one 1.2–1.3× longer than wide. Mesosoma length 1.3–1.4× height. Fore wing 4.8–5.2 mm in length, 1-R1 / length of pterostigma = 1.2–1.5, r / 3-SR = 2.2–3.6. Hind wing with 1r-m / 2-SC+R = 0.8–1.0. Hind leg: femur 4.6–5.2× longer than wide, length of femur: tibia: basitarsus = 1: 1.3–1.4: 0.6. First metasomal tergite 5.9–6.3× longer than its apical width.

**Male.** Unknown.

#### Distribution.

Japan (Hokkaidô; Honshû: Nagano Prefecture; Shikoku: Tokushima Prefecture).

#### Hosts.

Unknown.

#### Remarks.

This species closely resembles *A.rutilipes* Papp (Fig. 6) but differs in having the stouter penultimate antennal segment (in *rutilipoides*, 1.2–1.3× longer than wide (Fig. 7E), in *rutilipes* 1.8× (Fig. 6C)), the face finely punctate laterally, transversely punctate-rugose medially (finely punctate in *rutilipes*), the apical hyaline area of the fore wing not reaching the apico-posterior edge (Fig. 7G) (in *rutilipes*, reaching the apico-posterior edge (Fig. 6A)) and the metasoma reddish brown (Fig. 7A) (dark brown in *rutilipes* (Fig. 6A)). This species also resembles *A.dubius* Belokobylskij, but differs in having the antenna gradually darkened towards the apex (Fig. 7A) (in *dubius* uniformly reddish brown, not darkened towards the apex (Fig. 1A)), the penultimate antennal segment stouter (in *rutilipoides*, 1.2–1.3× longer than wide (Fig. 7E), in *dubius* 1.6–1.7× (Fig. 1E)), the face finely punctate laterally, transversely punctate-rugose medially (finely punctate in *dubius*), the malar suture indistinct or absent (distinct in *dubius*) the apical hyaline area of the fore wing comparatively large, almost reaching the apex of the marginal cell (Fig. 7G) (in *dubius* comparatively small, not reaching the apex of the marginal cell (Fig. 1F)), the vein m-cu of the fore wing antefurcal (Fig. 7G) (slightly postfurcal to interstitial in *dubius* (Fig. 1F)), and the hind femur slenderer, 4.6–5.2 times as long as wide (Fig. 7H) (4.1–4.3 times as long as wide in *dubius* (Fig. 1G)). This species is also similar to *A.ussuriensis* Belokobylskij, 1981 described from the Russian Far East, but differs in having the vertex densely punctate (vertex with distinct transverse striation in *ussuriensis*), the mesosoma black (mesosoma red except for the prothorax black in *ussuriensis*) and the metasoma entirely yellowish brown (metasoma black except for the red first metasomal tergite in *ussuriensis*).

## ﻿Discussion

Among the six Japanese species, *A.egregius* and *A.rufotestaceus* are widely distributed in the Palaearctic region, but four other species (*A.dubius*, *A.elasmuchae*, *A.flavicans*, and *A.rutilipoides* sp. nov.) are virtually confined to East Asia (China, Japan, Korea, the Russian Far East and Taiwan). Two Japanese species, *A.rutilipoides* sp. nov. and *A.dubius*, have a comparatively larger body and the fore wing with two fuscous bands and belong to a species complex with *A.rutilipes* from continental China, Korea, and Taiwan, and *A.ussuriensis* from continental China, Korea, and the Russian Far East. A comprehensive study on the fauna and phylogeny of *Aridelus* in and around East Asia is required.

All previously known host families of *Aridelus* (Acanthosomatidae, Pentatomidae, Plataspidae, Scutelleridae) belong to the superfamily Pentatomoidea (Shaw et al. 2001), but our study has revealed that *Aridelus* can also use the superfamily Coreoidea (including Alydidae) as host insects. While the most widely distributed species, *A.egregius*, is known to parasitize three host families, other Japanese species so far only one host family is known (Yu et al. 2016; present study). The host specificity of *Aridelus* species is an interesting problem that deserves further study.

## Supplementary Material

XML Treatment for Aridelus

XML Treatment for Aridelus dubius

XML Treatment for Aridelus egregius

XML Treatment for Aridelus elasmuchae

XML Treatment for Aridelus flavicans

XML Treatment for Aridelus rufotestaceus

XML Treatment for Aridelus rutilipoides

## Supplementary materials

Supplementary material 1 Table S1 Data type xlsx file Explanation note Comparison of Chinese specimens of *Aridelusrufotestaceus* Tobias and *A.rufiventris* Luo & Chen with Japanese and Korean specimens of *A.rufotestaceus*. https://binary.pensoft.net/file/669148This dataset is made available under the Open Database License (http://opendatacommons.org/licenses/odbl/1.0/). The Open Database License (ODbL) is a license agreement intended to allow users to freely share, modify, and use this Dataset while maintaining this same freedom for others, provided that the original source and author(s) are credited.Shunpei Fujie, Kaoru Maeto
